# Role of graphene oxide in mitigated toxicity of heavy metal ions on *Daphnia magna*†

**DOI:** 10.1039/c8ra09135h

**Published:** 2018-12-12

**Authors:** Lingfeng Ni, Yi Li

**Affiliations:** Key Laboratory of Integrated Regulation and Resource Development of Shallow Lakes, Ministry of Education, College of Environment, Hohai University Nanjing 210098 P. R. China envly@hhu.edu.cn +86-25-83786251 +86-25-83786251; State Key Laboratory of Pollution Control and Resources Reuse, Shanghai Institute of Pollution Control and Ecological Security, College of Environmental Science and Engineering, Tongji University Siping Road Shanghai 200092 P. R. China

## Abstract

Graphene oxide (GO) is increasingly used and inevitably released into aquatic environments, facilitating its interaction with traditional pollutants such as heavy metal ions. However, the potential effect of GO on the toxicity of heavy metal ions to aquatic animals is unknown. This work aims to assess the toxicity of heavy metal ions (Cu(ii), Cd(ii), and Zn(ii)) on *Daphnia magna* (*D. magna*) in the presence of GO. GO nanoparticles remarkably reduced the concentrations of heavy metal ions by adsorption and decreased the metal accumulation in *D. magna*. The maximum desorption rate of heavy metal ions from metal-adsorbed GO was below 5%. At pH 7.8, with addition of 2 mg L^−1^ GO, the 72 h median lethal concentration (LC_50_) values of Cu(ii), Cd(ii), and Zn(ii) were increased from 14.3, 38, and 780 μg L^−1^ to 36.6, 72, and 1010 μg L^−1^, respectively. The analyses of oxidative stress indicators suggested that the oxidative damage to *D. magna* by heavy metal ions was reduced after addition of GO at pH 7.8. Moreover, a higher pH level in the growing range (6.5 to 8.5) of *D. magna* led to weaker toxicity of metal ions with GO addition due to more adsorption and less bioaccumulation. The results revealed the role of GO nanoparticles in the mitigated toxicity of heavy metal ions in the aquatic environment.

## Abstract 2

Graphene oxide nanoparticles mitigates the biotoxicity of heavy metal ions (Cu(ii), Cd(ii), and Zn(ii)) on aquatic animals (*Daphnia magna*).

## Graphical abstract

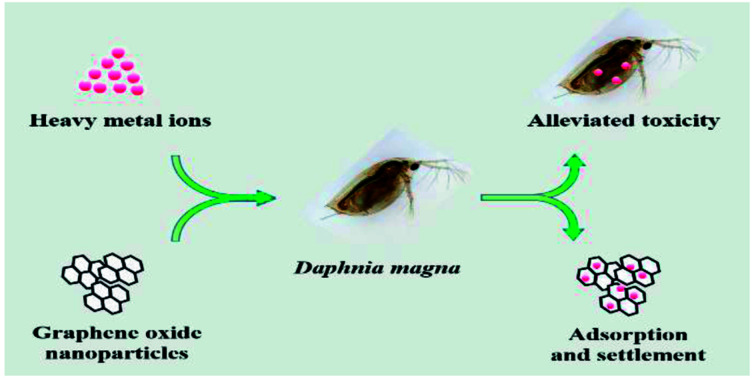

## 1 Introduction

Since the first isolation in 2004,^1^ graphene and its derivatives have brought tremendous improvement and development in diverse fields, such as nanoelectronics, catalysis and nanomedicine due to their exceptional mechanical, electronic, optical and catalytic properties.^2–4^ As an important intermediate product, graphene oxide (GO) could be used to directly produce graphene-based composites, resulting in its mass usage in the graphene industry.^5^ During production and application, GO will possibly find its way into the environment in the form of nanoparticles because of its good dispersity in most solvents.^5,6^ Deep evaluations on cell damage and bacterial toxicity of this new nanomaterial have been carried out in the last few years.^7–9^ For example, reduced GO was found to alter plant physiology and soil bacterial community composition in a rice-soil-bacterial ecosystem in the study of Hao *et al.*^10^ However, little attention was focused on its risks towards aquatic systems, which was probably due to its low toxicity to aquatic organisms. For example, the 48 h median lethal concentration (LC_50_) of GO on *Amphibalanus amphitrite* was very high (560 mg L^−1^) and GO merely reduced its swimming speed and settling.^11^

Once released into the aquatic environment, GO would possibly interact and co-exist with some background toxic substances.^5^ Due to weak hydrophobic interactions, the negatively charged surfaces of GO are favorable to interact with both organic and inorganic cations through electrostatic attractions.^12,13^ GO could remove and recover conventional pollutants such as heavy metal ions as carriers due to its large surface area, pore size and abundant oxygen-containing functional groups (*e.g.*, epoxy and hydroxyl groups).^14–16^ Therefore, the GO nanoparticles dispersed in the aquatic environment may reduce the concentration, change the existential state, and eventually affect the biotoxicity of heavy metal ions by adsorption. For example, as reported by Hu *et al.*, the antagonistic effects between GO and Cu(ii) reduced the ecotoxicity of Cu(ii) on *Scenedesmus obliquus*.^17^ However, study on the joint toxicity of GO and heavy metal ions on aquatic organisms, especially aquatic animals, is still limited and the toxic mechanism is unclear. The impact assessment of emerging nanoparticles (GO) on the biotoxicity of conventional pollutants (heavy metal ions) is urgently needed.

It was widely considered that, nanomaterials can obviously influence the biotoxicity of heavy metal ions. For example, as a common nanomaterial, TiO_2_ was found to enhance Cu(ii) toxicity to *Daphnia magna* (*D. magna*) by increasing the bioaccumulation of Cu(ii).^18^ On the contrary, in the water containing low DOC (dissolved organic carbon) concentration, TiO_2_ was found to reduce Cu(ii) toxicity to *D. magna* because of decreased Cu(ii) concentration in the water column and the sedimentation of Cu-adsorbed TiO_2_.^19^ Similarly, TiO_2_ was also found to alleviate Cd(ii) toxicity on rice seedlings by reducing the Cd(ii) uptake and distribution in roots and leaves.^20^ Therefore, concentrations variation and bioaccumulation of heavy metal ions induced by adsorption may be important factors of the joint toxicity between nanomaterials and heavy metal ions. Furthermore, heavy metal ions have been reported to induce oxidative damage in animals.^21,22^ So it is essential to study the effect of nanomaterials on heavy metal ions-induced oxidative damage as important toxicity mechanism. It was also reported that, traditional carbon nanomaterials (carbon nanotubes and *n*C_60_) increased the toxicity of heavy metal ions in *D. magna* by adsorbing metal ions and increasing metals accumulation as carriers.^23,24^ Although GO is also a kind of carbon nanomaterial, its effect on biotoxicity of heavy metal ions may be a totally different case. According to Yu and Wang, although the physical properties of different carbon nanomaterials were similar in some ways, their influences on metals accumulation and biotoxicity depend largely on the chemical properties.^25^ So the study of joint toxicity between GO and heavy metal ions became necessary due to the difference of GO from these traditional carbon nanomaterials in microscopic structure and surface functional groups.

In the present study, we examined the potential effects of GO on the biotoxicity of heavy metal ions to *D. magna*, a sensitive and standard *Crustacea* used in ecotoxicity experiments. Cu(ii), Cd(ii), and Zn(ii) were selected as representative heavy metal ions in three different levels of toxicity.^18,26,27^ The acute toxicity and bioaccumulation of these cations in the presence and absence of GO were compared to investigate the potential influence of GO nanoparticles on the metal ions. Oxidative stress indicators including the activities of superoxide dismutase (SOD), the content of malondialdehyde (MDA) and glutathione (GSH) were used to evaluate the oxidative damage in *D. magna* indirectly.^28^ In addition, the effect of pH values on the interaction of GO and heavy metal ions was also assessed, because pH determines the surface charge of nanoparticles and ultimately affects the nanoparticles' agglomeration dynamics and the adsorption of metal ions.^29,30^

## 2 Materials and methods

### 2.1 Chemicals and organisms

GO nanoparticles were provided by Hengqiu Graphene Technology Co., Ltd. (China) with ≥99.5% purity, 1.0–1.77 nm of thickness, about 0.2–5 μm of diameter, with 1–5 layers, and around 300–450 m^2^ g^−1^ of theoretical surface area. Prior to each experiment, bare GO stock suspension was prepared by adding GO nanoparticles to the culture medium of *D. magna* to reach a concentration of 2 mg L^−1^ and then ultrasonicated (35 kHz frequency) for 2 h assuring a homogeneous solution. Analytical-grade CuCl_2_·2H_2_O (≥99.0%), CdCl_2_·2.5H_2_O (≥98.0%), and ZnCl_2_ (≥99.0%) were obtained from Sinopharm Chemical Reagent Co., Ltd., (China). Bare metal ions solutions were diluted to target concentrations and stirred for 15 min. GO and metal ions mixed solutions were prepared by diluting high-concentration metal ions solutions into 2 mg L^−1^ GO suspension. To simplify the expression, the respective ions are referred as Cu(ii), Cd(ii), and Zn(ii) throughout the study. In general, the environmentally relevant pH values for *D. magna* is between 6.5 and 8.5. Therefore, test mediums were adjusted by additions of HCl and NaOH at pH 7.8 and 6.8 to study the impact of pH values. The change of ionic compositions, which was faintly influenced by the adjustment of pH using HCl and NaOH, was not further considered.

*D. magna* were cultured in permanent climate chamber (Ningbo Safe Experimental Instrument Co., Ltd, China) at 20 ± 1 °C with 16 : 8 (light: dark) photoperiod (800–1000 lx).^31^ The culture medium was prepared with NaHCO_3_ (0.096 g L^−1^), CaSO_4_·2H_2_O (0.06 g L^−1^), MgSO_4_ (0.06 g L^−1^), KCl (0.004 g L^−1^), and Na_2_SeO_4_ (2 μg L^−1^) according to the EPA standard method.^32^ The culture medium was changed three times a week to keep a relatively clean aquatic environment. *D. magna* were fed twice a day with *Scenedesmus obliquus* at the concentration of 1 × 10^5^ to 2 × 10^5^ cells per mL. Juvenile *D. magna* of 5 day-old after three generations of parthenogenesis were used in all experiments.

### 2.2 Characterization of GO

GO nanoparticles after ultrasonication in reaction medium were characterized by transmission electron microscopy (TEM, JEM-2010, JEOL, Japan). Fourier transform infrared (FTIR) technique was also used for the analysis of surface functional groups of GO. The spectrum was measured using an FTIR spectrometer (Bruker-Tensor 27, Germany) equipped with a KBr beam splitter (KBr, FTIR grade). Spectra were acquired in the 4000–400 cm^−1^ wave number with a 4 cm^−1^ resolution. About 2 mg of freeze–dried sample was pressed with 100 mg KBr to form pellets. Prior to use, the spectrum of KBr was used as blank. The surface area of GO was evaluated by a Micromeritics ASAP 2020M + C accelerated surface area analyzer.

### 2.3 Adsorption–desorption tests

The adsorption capacity of heavy metal ions onto GO nanoparticles was characterized with sorption experiments, aiming to guide the preparation of proper concentrations of metal ions and GO for the toxicity tests. By preliminary experiments, proper GO concentration was determined to be 2 mg L^−1^. The adsorption isotherms were obtained by varying the initial concentrations of Cu(ii) (5–80 μg L^−1^), Cd(ii) (10–100 μg L^−1^), and Zn(ii) (250–1500 μg L^−1^) at a fixed GO concentration (2 mg L^−1^). Firstly, the mixed solutions of GO and heavy metal ions were prepared and shaken for 72 h. The supernatants were then collected by centrifuging for 10 min at 12 000 rpm using a versatile compact centrifuge (Himac CF 16RX, HitachiCo., Ltd., Tokyo, Japan). The residual ions concentrations in the supernatants were measured by inductively coupled plasma mass spectrometry (ICP-MS; Xseries II, Thermo Fischer Scientific, Dreieich, Germany). The adsorbed heavy metal ions concentrations were calculated assuming mass balance between the initial and the final solutions. After the adsorption experiments, further experiments were conducted to investigate the ions desorption process from GO in the gut of *D. magna* as detailed in the ESI.† All adsorption and desorption tests were replicated three times, and only the average values were reported. The adsorption rates and desorption rates were calculated from eqn (A.1)–(A.3).A.1
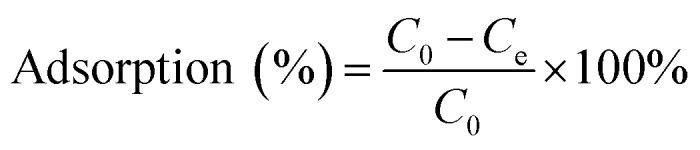
A.2
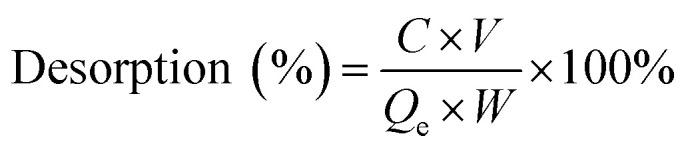
A.3
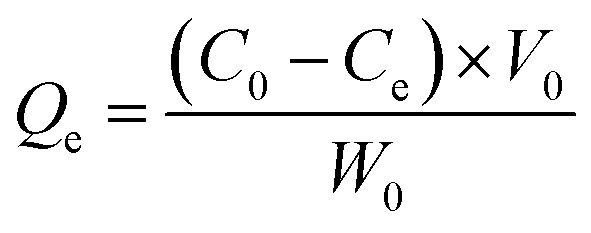
where *C*_0_ and *C*_e_ are, the metal ion concentration before and after adsorption (μg L^−1^), respectively; *C* is the ion concentration of the supernatant in desorption tests (μg L^−1^); *V* is the volume of the supernatant in desorption tests (L); *V*_0_ is the initial volume of the solution containing metal ions (L); *Q*_e_ is the adsorption capacity (μg mg^−1^); *W*_0_ is the initial weight of the adsorbent (mg), while *W* is the weight of the adsorbent in desorption tests (mg).

### 2.4 Acute toxicity tests

Firstly, different concentrations of GO solutions (2, 10, 50, 100, 150, and 200 mg L^−1^) were prepared to test acute toxicity of bare GO to *D. magna*. Then, different concentrations of Cu(ii) (5–80 μg L^−1^), Cd(ii) (10–100 μg L^−1^), and Zn(ii) (250–1500 μg L^−1^) ions were used to investigate the bare toxicity of heavy metal ions. After that, the mixture solutions of heavy metal ions and GO (2 mg L^−1^) were used to investigate the combined toxicity. Sufficient *D. magna* of 5 day-old from the same generation with similar size were collected. These *D. magna* were fed 2 hours before the start of each test and no additional food was added during the exposure. The testing solutions (50 mL) were prepared in 100 mL beakers, after which 10 *D. magna* were added. All the test solutions were shaken in a shaker at 20 ± 1 °C to reduce aggregation. In 3 d exposure process, mortality of *D. magna* was noted at 24, 48, and 72 h to calculate LC_50_ values of each case. All acute toxicity tests were replicated three times. Soon after the each experiment, surviving *D. magna* were collected, rinsed, stored at −20 °C for biochemical analysis.

### 2.5 Accumulation experiment

The concentrations of Cu(ii), Cd(ii), and Zn(ii) applied in the accumulation experiments were set at 14.3, 38, and 780 μg L^−1^, respectively, namely LC_50_ values according to acute toxicity tests at pH 7.8. The experiments procedure was the same as the acute toxicity tests. Body burden of metals was measured according to the method proposed by Fan *et al.*^33^ All the test groups were conducted with abundant replicates so that enough mobile *D. magna* after exposure could be collected for further accumulation tests. After 3 days exposure, 50 mobile *D. magna* were removed from each medium and depurated in pure water for 1 min to remove toxicants on the body surface. The samples were dried at 80 °C overnight, weighed on a microbalance to calculate the dry weight and then digested in 69% HNO_3_ (Aristar grade) at 110 °C overnight. Concentrations of Cu(ii), Cd(ii), and Zn(ii) in the digested samples were subsequently determined by ICP-MS. All tests were replicated three times. Whole body burden of heavy metal ions was expressed as μg g^−1^ dry weight and calculated based on the dry weight of *D. magna*.

### 2.6 Analysis of oxidative stress markers

Biochemical parameters for oxidative stress, including the activity of superoxide dismutase (SOD) and the content of glutathione (GSH) and malondialdehyde (MDA) were determined using a Diagnostic Reagent Kit (Nanjing Jiancheng Bioengineering Institute, Nanjing, China). After the exposure to toxic materials, about 50 surviving *D. magna* from each test group were collected as one sample. In order to collect enough surviving samples, multiple toxicity tests were prepared and treatment groups at high toxicants concentrations were abandoned. Details of testing procedure are provided in the ESI.†

### 2.7 Statistical analysis

All tests were performed in triplicate and all data was expressed as means ± standard deviation (SD). The LC_50_ and the associated 95% confidence intervals (95% CI) were estimated from a tolerance distribution analysis using a probit model (TRAP; Toxicity Relationship Analysis Program, v.2.21, USEPA). Differences between treatments were tested for significance using one-way analysis of variance (ANOVA), and *p* < 0.05 was considered statistically significant.

## 3 Results and discussion

### 3.1 Characterization of GO

The TEM image of the dispersion morphology and nanostructure of GO nanoparticles in reaction medium is shown in Fig. S1.† Layered GO nanosheets with few wrinkles were observed and GO nanoparticles were highly dispersed in reaction medium after ultrasonication. The oxygen-containing functional groups on the surface of GO nanoparticles were characterized by FTIR analysis (Fig. S2†). Different functional groups were found in the FTIR spectrum, *e.g.*, –OH group at 3407 cm^−1^, aromatic C=C group at 1623 cm^−1^, C–C in the carboxyl group at 1401 cm^−1^, C–O in the epoxide group at 1222 cm^−1^, and C–O in the alkoxy group at 1072 cm^−1^. These abundant oxygen-containing functional groups provided more opportunities for the interaction between heavy metal ions and GO. The specific surface area of GO was high at 188.68 m^2^ g^−1^ according to BET-N_2_ analysis, leading to excellent adsorption capacity for GO nanoparticles.

### 3.2 Adsorption of metal ions on GO

**Fig. 1:**
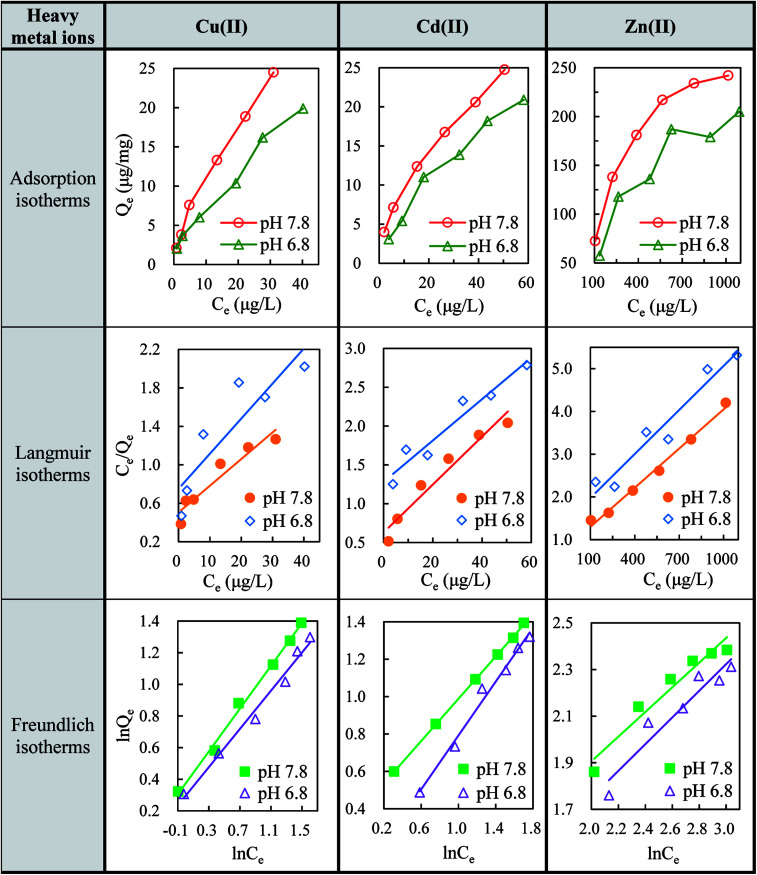
Adsorption isotherms, Langmuir isotherms and Freundlich isotherms for the adsorption of Cu(ii), Cd(ii) and Zn(ii) onto GO (2 mg L^−1^) at pH 7.8 and 6.8.

The sorption equilibrium tests provided the interactions of heavy metal ions with GO nanoparticles and the change in metal uptake from the dissolved phase. Fig. 1 shows the adsorption capacities of Cu(ii), Cd(ii), and Zn(ii) onto GO at different pH values (7.8 and 6.8). As shown in Fig. 1, Cu(ii), Cd(ii), and Zn(ii) were apt to be adsorbed on GO nanoparticles and the adsorption capacity increased with increasing metal ions concentrations. At pH 7.8, the maximum adsorption rates for Cu(ii), Cd(ii), and Zn(ii) reached 78, 77, and 51.8%, respectively. Moreover, a lower adsorption capacity of GO was observed when pH of the solution was adjusted from 7.8 to 6.8, indicating a better adsorption performance of GO for heavy metal ions at alkaline environment.

The Langmuir isotherms (eqn (B.1)) and Freundlich isotherms (eqn (B.2)) were adopted to describe the adsorption behaviors of heavy metal ions onto GO. The linear equations are as follows:B.1
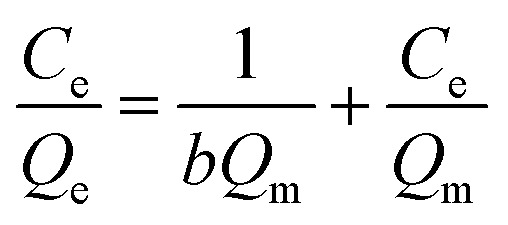
B.2
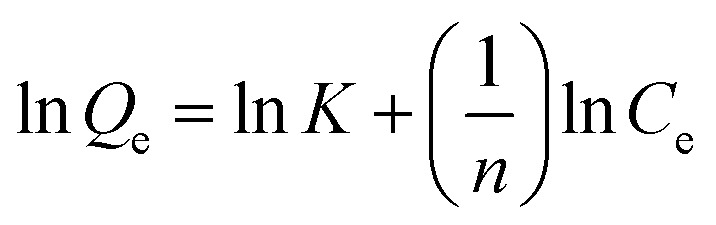
where *b* is a constant related to the free energy of adsorption (l μg^−1^), *Q*_m_ is the maximal adsorption capacity at equilibrium (μg mg^−1^), whereas *K* (L mg^−1^) and *n* is Freundlich constants related to the adsorption capacity and adsorption intensity, respectively. Normalized plots obtained from the Langmuir and Freundlich models are shown in Fig. 1. All isotherm parameters calculated from the plots are listed in Table 1. At pH 7.8, the maximum adsorption capacities for Cu(ii), Cd(ii), and Zn(ii) reached 36.1, 32.4, and 322.6 mg g^−1^, respectively. The high values of regression coefficient (*R*^2^) indicate a good agreement between the isotherm parameters and experiment values. The results of *R*^2^ showed that the adsorption of Cu(ii) and Cd(ii) were well fitted to Freundlich model. In addition, the Freundlich constant *n* is larger than 1, indicating favorable adsorption of Cu(ii) and Cd(ii) on GO under the studied conditions. However, in the case of Zn(ii), Langmuir isotherms fitted better with experimental data than Freundlich isotherms, in accordance with the study of Wang *et al.* that the Zn(ii) adsorption on GO maybe monolayer coverage.^34^

**Table 1:** The parameters for Langmuir and Freundlich isotherm models of Cu(ii), Cd(ii), and Zn(ii) adsorption onto GO (2 mg L^−1^) at pH 7.8 and 6.8

Metals	pH	Langmuir isotherms			Freundlich isotherms		
		*Q*_m_ (μg mg^−1^)	*b* (L μg^−1^)	*R* ^2^	*K* (μg mg^−1^)	1/*n*	*R* ^2^
Cu(ii)	7.8	36.101	0.055	0.913	2.357	0.676	0.995
	6.8	27.397	0.049	0.802	1.974	0.604	0.986
Cd(ii)	7.8	32.362	0.049	0.949	2.649	0.565	0.999
	6.8	37.594	0.021	0.939	1.178	0.720	0.988
Zn(ii)	7.8	322.581	0.003	0.992	7.032	0.529	0.943
	6.8	294.118	0.002	0.942	4.034	0.572	0.915

From the perspective of adsorption mechanism, the adsorption of metal ions on GO was attributed to complexation, ion-exchange, and electrostatic attraction.^35^ Irrespective of heavy metal ions speciation, the possible adsorption mechanism of divalent heavy metal ions on GO was as follows (M represents Cu, Cd, and Zn):

C.1 GO–COOH + M(ii) → GO–COO^−^–M(ii) + H^+^

C.2
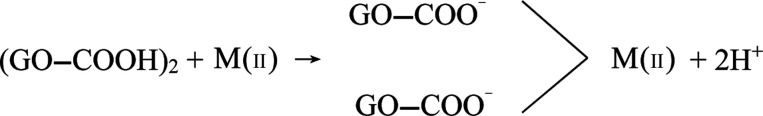

C.3 GO–OH + M(ii) → GO–O–M(ii) + H^+^

C.4
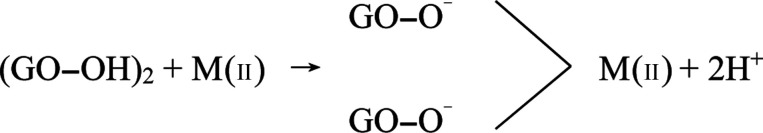


The cation-π bonds between the GO π-electrons and easily protonated metal ions were mainly responsible for the adsorption of ions onto GO surfaces.^5^ The abundant oxygen-containing functional groups on highly-dispersive GO nanosheets made the adjacent oxygen atoms available to bind metal ions. These groups could facilitate interstitial diffusion of metal ions to GO due to their hydrophilicity and water permeability.^13^ Therefore, GO can effectively concentrate Cu(ii), Cd(ii), and Zn(ii) from the medium. It can be speculated that the biotoxicity of heavy metal ions would be largely affected due to the reduction of free metal ions concentrations after the adsorption by GO nanoparticles. In addition, metal speciation or competing complexation reactions always render sorption capacity sensitive to changes in pH level. By affecting the deprotonation and speciation of the surface functional groups, pH could remarkably govern the sorption behaviors of metal ions. At higher pH value, the surface charge became more negative, leading to stronger electrostatic interactions and promoting the adsorption of metal ions (Fig. 1).

The desorption behaviors of heavy metal ions from GO nanoparticles at pH 7.8 were shown in Fig. S3.† The eluted proportions followed the order of Zn(ii) > Cd(ii) > Cu(ii), consistent with the affinity constants between GO and heavy metal ions.^36^ It was reported that, the desorption of heavy metal ions from GO was most effective under acidic conditions and much less in neutral water or alkaline conditions.^37^ Consequently, the maximum desorption percentage of the three heavy metal ions was below 5%, indicating the difficulty for heavy metal ions to be desorbed from GO nanoparticles in our test medium.

### 3.3 Effects of GO on metal ions accumulation

The microscopic picture of a *D. magna* after 72 h exposure to a 2 mg L^−1^ GO solution was shown in the ESI (Fig. S4†). According to the picture, little GO nanoparticles were ingested in the gastrointestinal tract of *D. magna*, but most of them were aggregated into small flocs and adhered in thoracic appendage and on external surface, from which GO nanoparticles were easy to be cleared or depurated. The GO flocs in the thorax may affect the normal operation of the thoracic appendage and hinder the feeding of *D. magna*. The accumulation of metal ions in *D. magna* with or without GO addition was investigated by batch experiments (Table 2). At pH 7.8, body burden of Cu(ii), Cd(ii), and Zn(ii) without GO in *D. magna* were 11.8, 36.3, and 20.9 folds to the control (*p* < 0.05), respectively, indicating that large amounts of heavy metal ions were accumulated in *D. magna*. With addition of GO (2 mg L^−1^), body burden of Cu(ii), Cd(ii), and Zn(ii) in *D. magna* decreased 26.8%, 31.6%, and 16.5%, respectively, at pH 7.8. Significant decreases (*p* < 0.05) of body burden of Cu(ii) and Zn(ii) were observed with GO addition compared with the case without GO at pH 7.8. Moreover, the accumulation of metals in all test groups was increased when pH value of test mediums was adjusted from 7.8 to 6.8.

**Table 2:** Bioaccumulation of heavy metal ions (μg g^−1^ dry weight) (mean ± SD; *n* = 3) with and without GO (2 mg L^−1^) in *D. magna* at pH 7.8 and 6.8 after 72 h exposure

Treatments	Cu(ii) (μg g^−1^)		Cd(ii) (μg g^−1^)		Zn(ii) (μg g^−1^)	
	pH 7.8	pH 6.8	pH 7.8	pH 6.8	pH 7.8	pH 6.8
Control	25 ± 2.1	29 ± 4.3	2.7 ± 0.4	2.1 ± 0.1	113 ± 13.4	117 ± 7.8
Metal (LC_50_)	295 ± 21.5	316 ± 33.7	98 ± 18.3	115 ± 11.4	2360 ± 65.4	2500 ± 76.2
Metal (LC_50_) with GO	216 ± 15.9	242 ± 22.0	67 ± 10.5	84 ± 5.7	1970 ± 35.3	2150 ± 27.4

Nanoparticles were reported to alter the metal bioaccumulation in two ways. In one aspect, they could adsorb large amounts of metal ions on their surface, reduce the free ions concentration, and decrease the bioaccumulation by aggregation and settlement of metals-adsorbed nanoparticles. In another aspect, the metals-adsorbed nanoparticles could be ingested by aquatic organisms and increase the bioaccumulation.^38^ In contrast with our results, Tao *et al.* demonstrated that the accumulation of copper in *D. magna* was enhanced in the presence of fullerene nanocrystal (*n*C_60_) at low concentration.^23^ Similarly, the addition of nontoxic concentration of single-walled carbon nanotubes (SWNTs) could enhance the uptake of copper in *D. magna* and multiwalled carbon nanotubes (MWCNTs) could increase the Ni accumulation in *D. magna*.^24,39^ Apparently, compared with these conventional carbon nanomaterials (*n*C_60_, SWNTs, and MWCNTs), GO played a totally different role in influencing the accumulation of heavy metal ions in *D. magna*.

As reported by Yu and Wang, functionalized carbon nanotubes apparently had an elevated accumulation effect of Zn(ii) and Cd(ii) as compared to non-functionalized carbon nanotubes due to rich carboxyl groups, which were capable of adsorbing metal ions.^25^ Therefore, the abundant oxygen-containing functional groups on GO were expected to play important roles in changing body burden of heavy metal ions in *D. magna*. Among carbon nanomaterial adsorbents such as CNTs, C_60_ and activated carbon (AC), GO nanoparticles possess the highest sorption capacity.^16,40^ Thus excellent affinity of GO to heavy metal ions substantially reduced metal ions concentrations in solutions. Meanwhile, heavy metal ions promoted the aggregation of GO nanoparticles in water columns. In the presence of divalent cations (Cu(ii), Cd(ii), and Zn(ii)), three types of cross-linking interactions may cause the aggregation of GO nanoparticles: (1) bridging the edges of the GO through chelating carboxylate groups, (2) intercalating between the basal planes through either weak alkoxide or dative bonds from carbonyl and hydroxyl groups, and (3) cross-linking of the hydrogen bonds formed among the oxygen functional groups on GO surfaces and the inter lamellar water molecules.^41,42^ Therefore, the metals-adsorbed GO nanoparticles were apt to aggregate and settle to bottom of the beaker, decreasing the chance of being filter-fed by *D. magna*. In summary, GO largely decreased free metal ions concentrations and was rarely ingested by *D. magna*, resulting in decreased bioaccumulation of heavy metal ions. Besides, the amount of accumulated metal ions varied at different pH levels. The accumulation of Cu(ii), Cd(ii), and Zn(ii) in *D. magna* at pH 7.8 was lower than that at pH 6.8 (Table 2). Higher pH value (7.8) increased the adsorption capacity of GO (Fig. 1) and may promote the aggregation of the metals-adsorbed GO nanoparticles.

### 3.4 Effects of GO on toxicity of metal ions

**Fig. 2:**
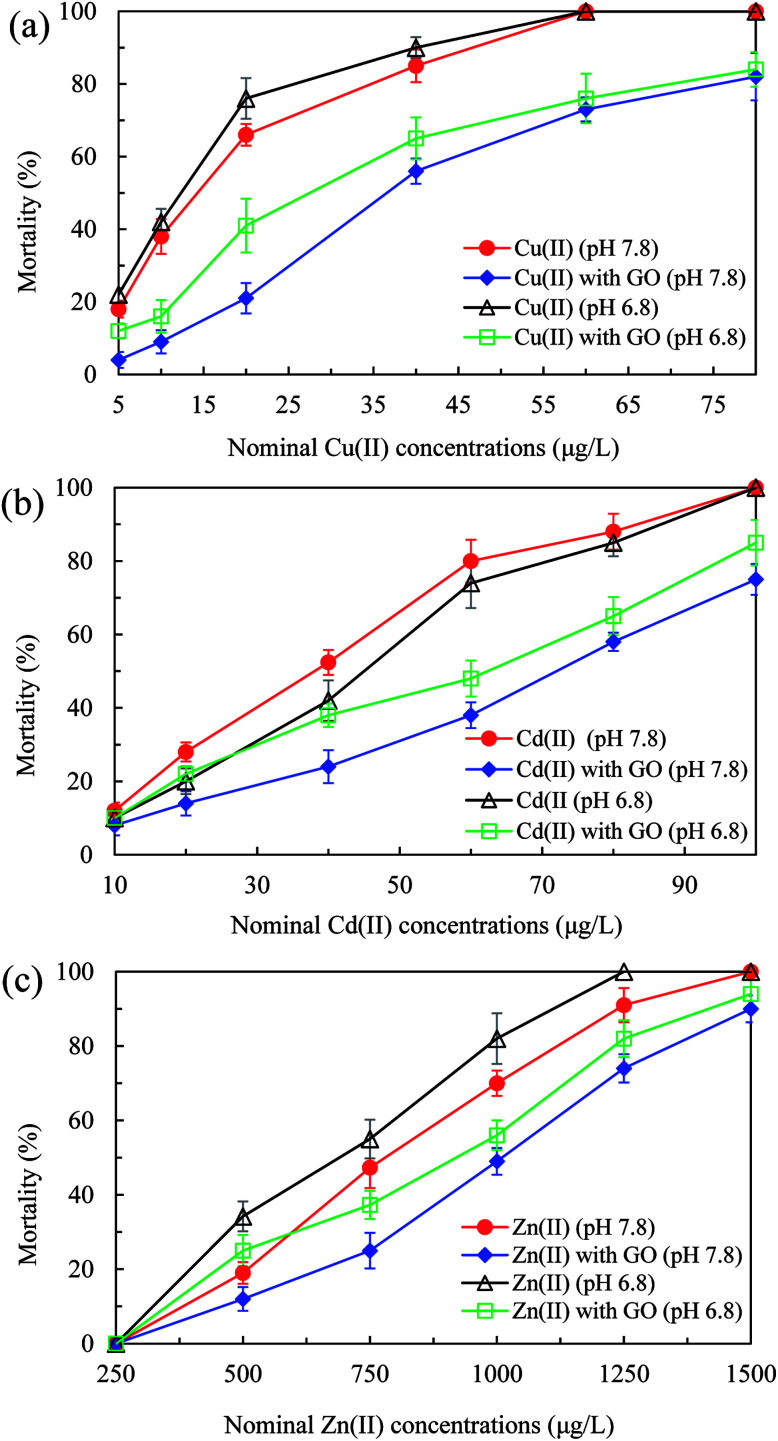
Mortality of *D. magna* in various concentrations of Cu(ii) (a), Cd(ii) (b) and Zn(ii) (c) with and without GO (2 mg L^−1^) at pH 7.8 and 6.8 after 72 h exposure.

First of all, the 72 h LC_50_ value of bare GO was determined to be 145 mg L^−1^ (process is not shown) and no mortality of *D. magna* was observed at low GO concentrations (<50 mg L^−1^), in accordance with the study of Zhao *et al.*^5^ Therefore, the GO concentration at 2 mg L^−1^ used in our tests could be regarded as nontoxic to *D. magna*. As shown in Fig. 2, mortalities of *D. magna* in all test groups increased with increasing heavy metal ions concentrations. In the presence of GO, mortalities of *D. magna* decreased obviously after exposure to Cu(ii), Cd(ii), and Zn(ii) compared to the case without GO. The maximum mortality decrease reached 45, 42, and 22.3% for Cu(ii) (20 μg L^−1^), Cd(ii) (60 μg L^−1^), and Zn(ii) (750 μg L^−1^), respectively, at pH 7.8. By addition of GO, the 72 h LC50 values of Cu(ii), Cd(ii) and Zn(ii) at pH 7.8 increased from 14.3, 38, and 780 μg L^−1^ to 36.6, 72, and 1010 μg L^−1^, respectively, suggesting that the toxicity of heavy metal ions was mitigated. In addition, with addition of GO, mortalities of *D. magna* in all test groups were increased by decreasing pH value of test mediums from 7.8 to 6.8.

As reported by Nowack and Bucheli, the interaction of nanoparticles with toxic pollutants can both amplify and alleviate the toxicity.^43^ Nanoparticles could mitigate the toxicity by adsorbing the pollutant and reducing its free concentration. But if the pollutant-loaded nanoparticles are taken up by organisms, the toxicity could be amplified. The study of Rosenfeldt *et al.*, which reveals the reduction of copper toxicity by nanosized TiO_2_, proposed the same viewpoint that TiO_2_ nanoparticles substantially reduced Cu(ii) in the water column and decreased the uptake of Cu(ii) in *D. magna* by agglomeration.^19^ In this study, we conclude that, whether the toxic effect is aggravated or mitigated lies on three elements: the adsorption degree of pollutants on nanoparticles, the uptake amount of pollutants-loaded nanoparticles, and the desorption of pollutants from nanoparticles in the body of organisms.

In contrast with the results of our study, SWNTs and *n*C_60_ were reported to enhance the toxicity of Cu(ii) and MWCNTs could enhance the toxicity of Ni in *D. magna*.^23,24,39^ For explaining these different results from ours, MWCNTs were selected as examples to compare with GO (Table 3). The most dominant adsorption mechanisms of metals onto carbon nanomaterials are physical adsorption and chemical interaction.^44,45^ The former depends mainly on the specific surface area and the later depends mainly on the surface acidic functional groups or oxygen functionalities.^46^ As shown in Table 3, the specific surface area and surface acidic groups of GO were 2.02 and 20.33 times of those of MWCNTs, respectively. Therefore, better adsorption capacity of GO than MWCNTs could effectively reduce the metal ions concentrations in testing solutions and alleviate their biotoxicity by directly removing the toxicants. Moreover the desorption rates of Cu(ii), Cd(ii), and Zn(ii) from GO were lower than those from MWCNTs (Table 3). Poor desorption rates could decrease the release of heavy metal ions from ingested metals-adsorbed nanoparticles in *D. magna* and further decrease the biotoxicity. In addition, low toxicity of bare GO (LC_50_ at 145 mg L^−1^) compared with MWCNTs (LC_50_ at 2.48 mg L^−1^) for *D. magna* could minimize the joint toxicity with heavy metal ions. Taken together, the mitigated toxicity of heavy metal ions brought by GO was attributed to high adsorption and low desorption capacity, decreased bioaccumulation and weak bare GO toxicity.

**Table 3:** Comparison of GO and MWCNTs nanoparticles in physicochemical properties, adsorption and desorption capacity to metal ions, and biotoxicity to *Daphnia magna*

Carbon nanomaterials	Specific surface area (m^2^ g^−1^)	Surface acidic groups (mmol g^−1^)	Adsorption capacity (mg g^−1^)			Desorption rate (%)			LC_50_ (mg L^−1^)
			Cu(ii)	Cd(ii)	Zn(ii)	Cu(ii)	Cd(ii)	Zn(ii)	
GO	188.68	1.24 (ref. 47)	36.1	32.4	322.6	2.3	3.2	4.6	145
MWCNTs	93.59 (ref. 47)	0.061 (ref. 47)	3.21 (ref. 48)	1.49 (ref. 49)	2.70 (ref. 50)	5.5 (ref. 37)	3.4 (ref. 37)	5.5 (ref. 37)	2.48 (ref. 51)

The interactions between GO and heavy metal ions and their biotoxicity to *D. magna* were also influenced by pH values. As Fig. 2 shows, compared with the case at pH 7.8, lower pH value (6.8) enhanced the mortalities of *D. magna* in the presence of GO by decreasing the adsorption (Fig. 1) and increasing the bioaccumulation (Table 2) of heavy metal ions. Moreover, metal ions were apt to desorb from metals-adsorbed GO in the body of *D. magna* at lower pH (Fig. S3†), giving rise to aggravated toxicity.^52^ In addition, without GO, the mortalities of *D. magna* by Cu(ii) and Zn(ii) at pH 6.8 were higher than that at pH 7.8 (Fig. 2). At lower pH value, the enhanced toxicity of Cu(ii) and Zn(ii) was probably due to the increase of free copper and zinc ions.^53,54^ In contrast, the toxicity of Cd(ii) at pH 6.8 was lower than that at pH 7.8, which could be speculated that some physiological reactions, such as metallothionein, within organisms were triggered to relieve the toxicity of Cd.^55^

### 3.5 Oxidative damage caused by metal ions with and without GO

**Fig. 3:**
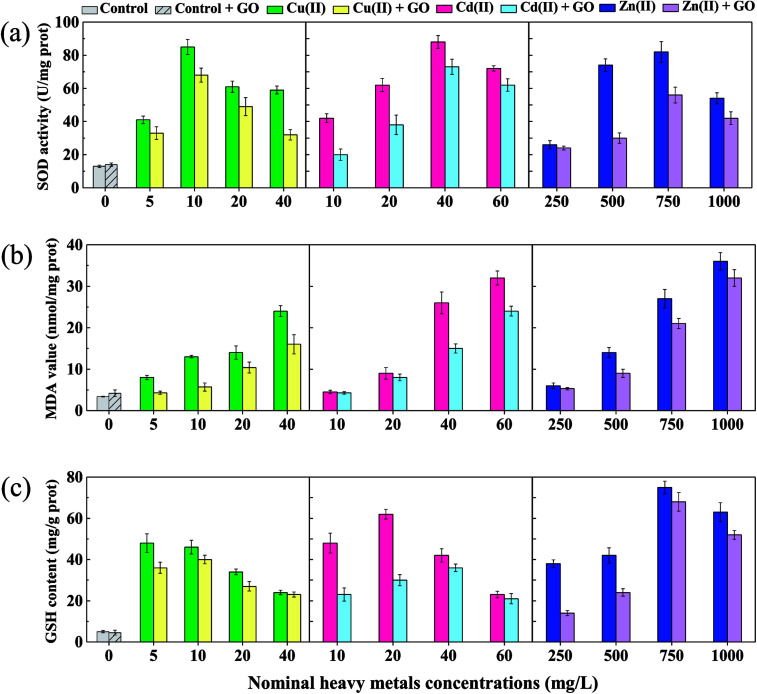
Effects of various concentrations of Cu, Cd, and Zn with and without GO (2 mg L^−1^) on superoxide dismutase (SOD) activities (a), malondialdehyde (MDA) levels (b), and reduced glutathione (GSH) contents (c) in *D. magna* after 72 h exposure.

Indicators of oxidative stress caused by heavy metal ions with and without GO addition are shown in Fig. 3. In the absence of GO, SOD activities treated with heavy metal ions increased significantly (*p* < 0.05) to maximum values of 6.5 (Cu(ii)), 6.8 (Cd(ii)), and 6.3 (Zn(ii)) times of the control, and then decreased with increasing the metal ions concentrations (Fig. 3a). It was reported that, cells in organisms are capable to deal with increased reactive oxygen species (ROS) up to a certain point without any deleterious effect on cellular function or viability.^28^ So the increase of SOD was due to the elimination of ROS by the self-protection mechanism and anti-oxidative stress in *D. magna*.^56^ But high concentrations of metal ions induced too much ROS, which went beyond the bearing limit of *D. magna* to remove oxygen radicals, leading to the reduction of SOD activity. Moreover, MDA levels increased significantly (*p* < 0.05) in a dose-dependent manner to maximum values of 7.1 (Cu(ii)), 9.4 (Cd(ii)), and 10.6 (Zn(ii)) times of the control in the absence of GO (Fig. 3b), suggesting that more and more lipid peroxidation had occurred and the oxidative damage became more serious in the body of *D. magna* with increasing ions concentrations.^57^ In addition, the variation trend of GSH content was observed similar to SOD activities (Fig. 3c) without GO. GSH is an important antioxidant and the amount of GSH could reflect the antioxidant potential of *D. magna*. Some antioxidant enzymes eliminates the oxygen radicals at the expense of GSH.^58^ The significant decrease (*p* < 0.05) of GSH contents at high Cu(ii) (40 μg L^−1^) and Cd(ii) (60 μg L^−1^) concentrations compared with the maximum values further proved the dose-dependent aggravation of oxidative damage, in accordance with the increased mortality of *D. magna* at high metals concentrations. Moreover, a slight decrease (*p* > 0.05) of GSH content for Zn(ii) at 1000 μg L^−1^ compared with the maximum value was also observed. Specifically, the maximum GSH contents for Cu(ii) and Cd(ii) were obtained at 5 and 20 μg L^−1^, respectively, while that was obtained at 750 μg L^−1^ for Zn(ii), indicating that Cu(ii) and Cd(ii) were more toxic and could cause more oxidative damage than Zn(ii) at the same concentration.

As shown in Fig. 3, the addition of GO decreased SOD activities, MDA values, and GSH contents in all test groups (except the control) compared with the case without GO, which possibly explained the mitigated toxicity of heavy metal ions to *D. magna*. A similar result was obtained in the study of Konate *et al.* that, Fe_3_O_4_ nanoparticles decreased the growth inhibition of heavy metals on wheat seedlings by reducing oxidative stress and the protective role was confirmed by the decrease in MDA content.^59^ The reduction of oxidative damage by GO was probably due to decreased concentrations of metal ions in water columns, reduced body burden, and weak desorption of metal ions from metals-adsorbed GO in the body of *D. magna*. In addition, no obvious oxidative stress reaction was observed in Fig. 3 when *D. magna* was exposed to bare GO, in accordance with the low acute toxicity of 2 mg L^−1^ GO. We can speculate that, low concentrations of GO nanoparticles may induce obvious oxidative damage at cellular level but not in organisms like *D. magna*.

## 4 Conclusions

This study reveals the role of graphene oxide (GO) nanoparticles in alleviated toxicity of heavy metal ions on *D. magna*. With GO addition, the 72 h LC_50_ values of Cu(ii), Cd(ii), and Zn(ii) were increased from 14.3, 38, and 780 μg L^−1^ to 36.6, 72, and 1010 μg L^−1^, respectively, at pH 7.8. The interaction between GO and heavy metal ions reduced the concentrations of metal ions in water columns by adsorption, decreased the bioaccumulation of heavy metal ions, and decreased the desorption of metal ions from GO in *D. magna*. The oxidative damage of heavy metal ions to *D. magna* was mitigated with the addition of GO by analysis of oxidative stress markers (SOD, GSH, and MDA). Moreover, lower pH value brought aggravated toxicity of heavy metal ions to *D. magna* in the presence of GO. Except for pH, studies on other influencing factors such as temperature and water hardness are still necessary in the following research. Chronic effect and cellular-level effect of GO on toxicity of heavy metal ions are also suggested to be studied.

## Conflicts of interest

There are no conflicts to declare.

## Supplementary Material

RA-008-C8RA09135H-s001
