# Supplementary material for: Role of graphene oxide in mitigated toxicity of heavy metal ions on Daphnia magna
Source: RSC Adv. 2018 Dec 12;8(72):41358–67. doi: 10.1039/c8ra09135h (PMC9091651; doi:10.1039/c8ra09135h)
Supplement: RA-008-C8RA09135H-s001 [file RA-008-C8RA09135H-s001.pdf]

Electronic Supplementary Material (ESI) for RSC Advances.
This journal is © The Royal Society of Chemistry 2018

Electronic supplementary information for

Role of Graphene oxide in mitigated toxicity of heavy metal ions on *Daphnia magna*[1]

Lingfeng Ni and Yi Li*

Key Laboratory of Integrated Regulation and Resource Development of Shallow Lakes, Ministry of Education, College of Environment, Hohai University, Nanjing 210098, P.R. China**.** E-mail: envly@hhu.edu.cn

Tel: 86-25-83786251; Fax: 86-25-83786251

Address: Ministry of Education Key laboratory of integrated regulation and resource development on shallow lakes, College of Environment, Hohai University, Nanjing 210098, P.R. China

Analysis of oxidative stress markers.

Biochemical analyses of oxidative stress indicators were performed at end of the *D. magna* toxicity tests.[1, 2] To collect enough mobile *D. magna* samples, groups treated with high toxicants concentrations were abandoned. *D. magna* samples from each test groups were stored at -20°C and treated according to the instructions given by Diagnostic Reagent Kits for oxidative stress parameters (Nanjing Jiancheng Bioengineering Institute, Nanjing, China). The samples were first defrosted, pooled and rinsed in pre-chilled 0.86% physiological saline. They were then homogenized on ice in a glass homogenizer containing physiological saline at approximately 1:10 (w/v) ratio. The homogenate was centrifuged at 10,000 rpm at 4°C for 10 min to obtain the supernatant for the enzyme activity assays. The supernatant fluid was collected and analyzed directly as enzyme sources for superoxide dismutase (SOD) activity, reduced glutathione (GSH) content and thiobarbituric acid (TBARS) reactive substances to assess lipid peroxidation.

SOD specific activities and GSH contents were determined by a UV-Vis spectrophotometer (HACH DR6000 LPV441.99.00002) at 550 nm and 412 nm, respectively. The SOD activity measurement was based on its ability to inhibit the oxidation of oxymine by superoxides produced by the xanthine/xanthine oxidase superoxide generation system. The red product (nitrite) produced by the oxidation of oxymine had absorbance at 550 nm. One unit of SOD activity (U) was defined as the amount of enzyme yielding 50% inhibition of nitrite formation in 1 mL of reaction solution and was expressed as Umg/g protein. GSH was studied as a non-enzymatic antioxidant. GSH concentrations were determined using 5, 5-dithionitrobenzoic acid and the results were expressed as mg/g protein. Lipid peroxidation products (measured as malondialdehyde) were quantified

based on the thiobarbituric acid (TBARS) method in which the formation of TBARS was detected at 532 nm. The malondialdehyde (MDA) concentration was expressed as nmol/mg protein. Protein content in the homogenates was assayed using the method of Bradford with bovine serum albumin as the standard.[3]

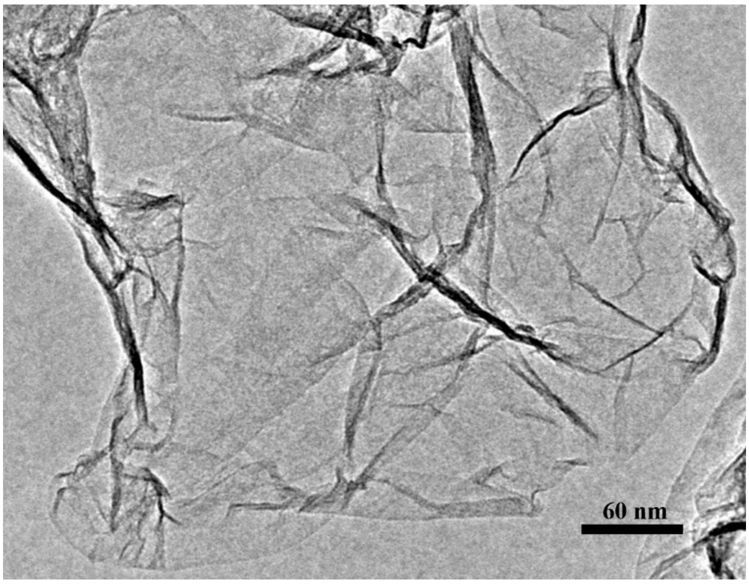

Fig. S1 TEM image of graphene oxide (GO) nanoparticles after ultrasonication in culture medium.

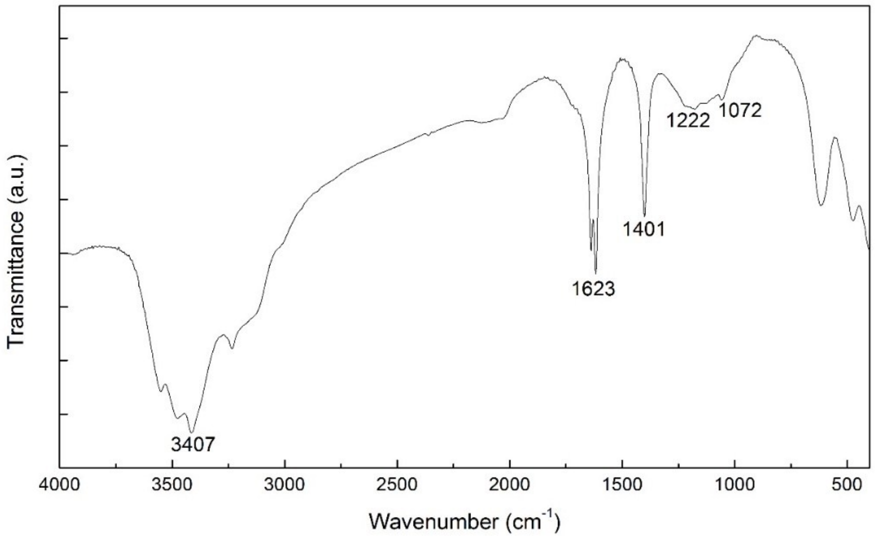

Fig. S2 FTIR spectra of graphene oxide (GO) nanoparticles.

Desorption of Heavy Metal Ions from GO.

After the adsorption experiments, the Cu(II), Cd(II) and Zn(II) adsorbed GO nanoparticles at pH 7.8 were separated using 0.22-μm membrane filters, then thoroughly rinsed with the culture medium, dried under vacuum and weighed. Since the pH in *D. magna* gut is neutral,[4] they were placed in a 100 mL beaker with 50 mL culture medium at pH 7.0 under stirring for desorption. The solutions were stirred for 12 h to ensure a full desorption equilibrium. After that, the supernatant was collected by centrifuging for 10 min at 12,000 rpm using a versatile compact centrifuge. The Cu(II), Cd(II) and Zn(II) concentrations in the supernatants were measured by ICP-MS. Results of desorption rates tests were shown in Fig. S3.

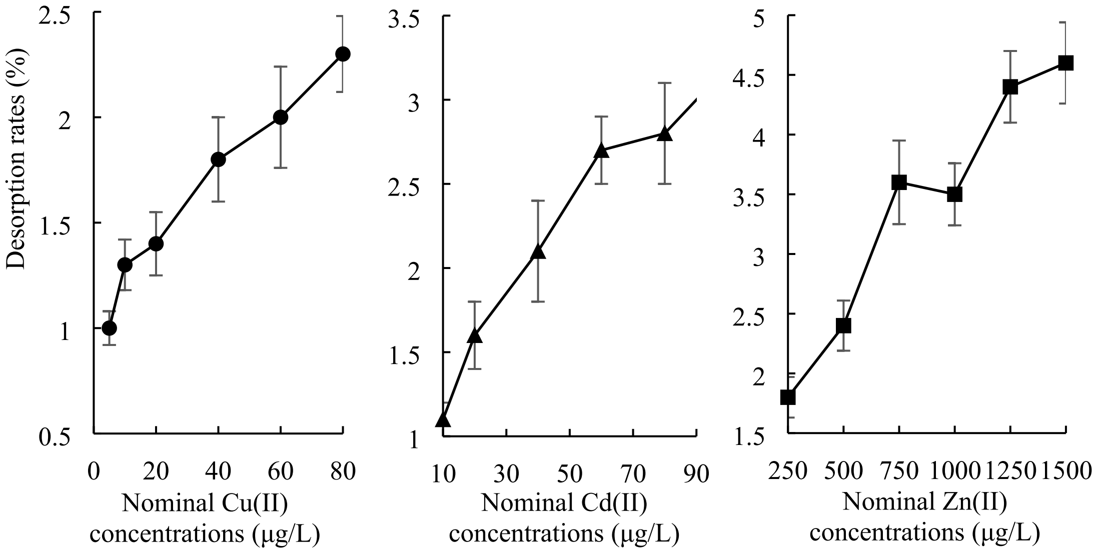

Fig. S3 Desorption rates of Cu(II), Cd(II) and Zn(II) (from left to right) from GO nanoparticles at pH 7.8.

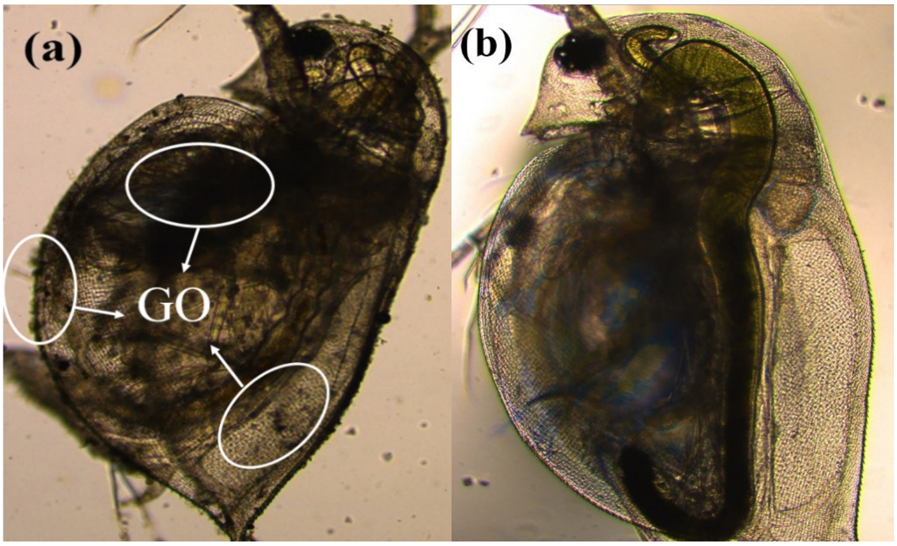

Fig. S4 Images of *D. magna* after 72 h exposure to GO (2 mg/L) (a) and control (b)

References

1. Y. Liu, J. S. Wang, Y. H. Wei, H. X. Zhang, M. Q. Xu and J. Y. Dai, *Aquat Toxicol*, 2008, **89**, 242-250.
2. C. Barata, I. Varo, J. C. Navarro, S. Arun and C. Porte, *Comp Biochem Phys C*, 2005, **140**, 175-186.
3. M. M. Bradford, *Anal Biochem*, 1976, **72**, 248-254.
4. R. W. Pennak, *Hydrobiologia*, 1955, **7**, 126-126.
